# Preclinical Bioavailability Assessment of a Poorly Water-Soluble Drug, HGR4113, Using a Stable Isotope Tracer

**DOI:** 10.3390/pharmaceutics15061684

**Published:** 2023-06-08

**Authors:** Eun Ji Ha, Jeong In Seo, Shaheed Ur Rehman, Hyung Soon Park, Sang-Ku Yoo, Hye Hyun Yoo

**Affiliations:** 1 Institute of Pharmaceutical Science and Technology, College of Pharmacy, Hanyang University, Ansan 15588, Gyeonggi-do, Republic of Korea; 2 Department of Pharmacy, Abasyn University, Peshawar 25000, Pakistan; 3 Glaceum Inc., Yeongtong-gu, Suwon 16675, Gyeonggi-do, Republic of Korea

**Keywords:** bioavailability, stable isotope, poorly water-soluble drugs, liquid chromatography–tandem mass spectrometry

## Abstract

Drug solubility limits intravenous dosing for poorly water-soluble medicines, which misrepresents their bioavailability estimation. The current study explored a method using a stable isotope tracer to assess the bioavailability of drugs that are poorly water-soluble. HGR4113 and its deuterated analog, HGR4113-d7, were tested as model drugs. To determine the level of HGR4113 and HGR4113-d7 in rat plasma, a bioanalytical method using LC-MS/MS was developed. The HGR4113-d7 was intravenously administered to rats that were orally pre-administered HGR4113 at different doses; subsequently, the plasma samples were collected. HGR4113 and HGR4113-d7 were simultaneously determined in the plasma samples, and bioavailability was calculated using plasma drug concentration values. The bioavailability of HGR4113 was 53.3% ± 19.5%, 56.9% ± 14.0%, and 67.8% ± 16.7% after oral dosages of 40, 80, and 160 mg/kg, respectively. By eliminating the differences in clearance between intravenous and oral dosages at different levels, acquired data showed that the current method reduced measurement errors in bioavailability when compared to the conventional approach. The present study suggests a prominent method for evaluating the bioavailability of drugs with poor aqueous solubility in preclinical studies.

## 1. Introduction

Bioavailability indicates the rate at and extent to which therapeutically active medicines enter systemic circulation and become accessible at their sites of action [1]. Thus, throughout the preclinical and clinical studies, its evaluation needs to be conducted. Pharmacokinetic parameters derived from bioavailability studies, such as the rate/extent of the absorption, distribution, metabolism, and elimination of tested drugs, are crucial for screening pharmacologically active intermediates and prodrugs, selecting optimal drug formulations and routes of administration, and developing appropriate dosing regimens [2].

In recent decades, novel therapeutics tend to have high molecular weight and high lipophilicity, causing poor water solubility [3,4,5]. Currently, it is estimated that over 90% of the compounds in the drug discovery stage are shown to have insufficient water solubility [6]. Oral drug administration is most prevalent; however, an oral dosage of a poorly soluble drug may decrease bioavailability because of slow or incomplete dissolution and possible precipitation in the gastrointestinal tract [7,8,9]. Traditionally, preclinical bioavailability studies have been conducted with intravenous and extravascular dosing in different types of animal models (rats, mice, and dogs) [10,11,12,13]. The total AUC was obtained from disparate administration routes, and absolute bioavailability (F) was determined using the following equation: F = (AUC_extravascular_ × Dose_intravenous_)/(AUC_intravenous_ × Dose_extravascular_). Here, it was assumed that the clearance was an integral component for accurate calculation [14]. Therefore, variation in clearance values between intravenous and extravascular routes may pull down the systematic accuracy in the bioavailability calculation. A drug’s low solubility is a major hurdle for increasing the maximum intravenous dosage as drugs must not precipitate in the bloodstream when administered intravenously [15]. Accordingly, the extravascular dose is generally higher than the intravenous dose, and drug plasma concentration levels between the two dosing routes may vary. This may cause a difference in clearance values for the two administration routes, resulting in bioavailability miscalculations [15,16].

HGR4113 ((R)-2-(8,8-dimethyl-2,3,4,8,9,10-hexahydropyrano[2,3-f]chromen-3-yl)-5-propoxyphenol) is a new drug candidate that is derived from glabridin. Glabridin is an isoflavan isolated from the root extract of licorice (*Glycyrrhiza glabra*) [17]. Glabridin has been extensively studied as a natural compound with known anti-oxidative and anti-inflammatory activities and an improving effect on metabolic dysregulation [18,19]. HGR4113 not only improves the chemical instability and metabolic characteristics found in glabridin but also enhances its efficacy. HGR4113 is currently in phase 1 clinical trials (NCT05642377) targeting type 2 diabetes, and it improves insulin sensitivity and blood glucose levels via its antioxidant and anti-inflammatory effects [20]. HGR4113 is a highly hydrophobic compound with a Log D value greater than 3.69, and its solubility is less than 0.2 μg/mL in water, indicating poor water solubility.

**Figure 1:**
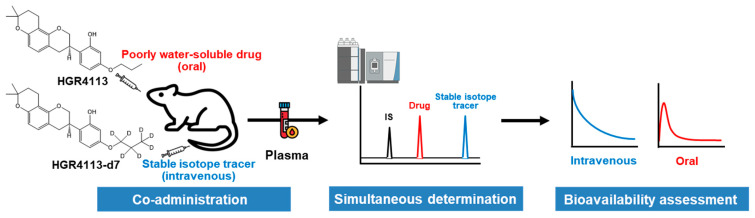
Scheme of the method for assessing the bioavailability of poorly water-soluble drugs using a stable isotope tracer.

In this study, the bioavailability of HGR4113 in rats was evaluated with a stable isotope tracer (HGR4113-d7) and LC-MS/MS (Figure 1 and Figure S1). With the isotopic approach, plasma drug concentrations for intravenous and oral doses can be evaluated from the same plasma sample, reducing variations from concentration-dependent clearance [15,16]. Herein, we demonstrate the usefulness of the isotopic approach to assess the preclinical bioavailability of new drug compounds lacking water solubility.

## 2. Materials and Methods

### 2.1. Chemicals and Reagents

The drugs used in this study, including HGR4113 ((R)-2-(8,8-dimethyl-2,3,4,8,9,10-hexahydropyrano[2,3-f]chromen-3-yl)-5-propoxyphenol), HGR4113-d7, and HSG4112 (internal standard, IS) (Figure S2) [21,22], were obtained from Glaceum Incorporation (Suwon, Reepublic of Korea). HPLC-grade MeOH (methanol), ACN (acetonitrile), and acetic acid were purchased from J.T. Baker (Phillipsburg, NJ, USA). Distilled water was purified using a Milli-Q system (Millipore, Bedford, MA, USA). Pooled rat liver microsomes were purchased from BD Gentest (Woburn, MA, USA). Glucose-6-phosphate, β-NADP+, and glucose-6-phosphate dehydrogenase were purchased from Milliporen Sigma (St. Louis, MO, USA). All other chemicals used in this study were analytical grade as received.

### 2.2. Animals

Male Sprague Dawley rats (270–310 g, eight weeks old), were acquired from OrientBio (Seongnam, Republic of Korea) and acclimatized for two days before commencing the experiments. Polycarbonate cages (one rat/cage) and a 12 h light/dark cycle were employed to keep the rats in an optimized environment of temperature (25 ± 2 °C) and humidity (50 ± 20%) during the study, with unrestricted access to food and drink.

### 2.3. Dosing Solution Preparation

The oral dosing solution of HGR4113 was prepared by dissolving 10% linoleoyl Polyoxyl-6 glycerides in medium-chain triglycerides solution (35% (*v*/*v*) of the whole solution) in a Falcon tube, followed by ultrasonic treatment for 10 min. This solution was mixed with 0.5% MC aqueous solution (65% (*v*/*v*) of the whole solution) and homogenized for at least three minutes at 30,000 rpm in a homogenizer (T25D, IKA, Staufen, Germany). The entire solution was sonicated for 30 min after being transferred to a glass vessel to obtain final concentrations of 10 or 20 mg/mL. An intravenous dosing suspension (0.75 mg/mL; HGR4113 or HGR4113-d7) was prepared in a mixture of phosphate-buffered saline and ethanol (1:1, *v*/*v*), followed by sonication for 10 min.

### 2.4. Animal Experiments

For the evaluation of kinetic isotope effects between HGR4113 and HGR4113-d7, four rats were intravenously administered 1 mg/kg mixture of HGR4113:HGR4113-d7 (1:1). Subsequently, retro-orbital plexus blood was collected using heparinized capillary tubes (Kimble Chase, NJ, USA) at 0.083, 0.25, 0.5, 0.75, 1, 2, 4, 6, and 8 h after drug administration. For the bioavailability test, the rats underwent blood sampling a day before the pharmacokinetic experiments. Rats were anaesthetized with a 2.5:1 *v*/*v* combination of Zoletil^®^ 50 (tiletamine-plus-zolazepam, Virbac, France) and Rompun^®^ (xylazine-HCl, Bayer Korea, Seoul, Republic of Korea). Polyethylene tubing (PE 50, Becton-Dickinson and Company, Franklin Lakes, NJ, USA) was implanted into the carotid artery of the anaesthetized rats. The PE 50 tubing was connected to a 1 mL syringe for sampling their blood conveniently with a fixture at the head and neck in order to not interrupt the movement of the rats throughout the experiment. The dose of the test compound was set to 40 (G1), 80 (G2), and 160 (G3) mg/kg for oral and 1 mg/kg for intravenous administration. After oral administration of HGR4113 to rats, HGR4113-d7 was administered intravenously at 3 h and 55 min, which is the expected time for oral T_max_ (approximately 4 h). The timetable for drug administration and blood collection is shown in Figure 4A. In heparinized microtubes, blood samples (about 200 µL) were collected and centrifuged at 4 °C for 5 min at 13,000× *g* and then stored at −20 °C before analysis. All animal procedures were approved by the Institutional Animal Care and Use Committee of Hanyang University (2020-0117A).

### 2.5. Calibration and Quality Control (QC) Standards

Stock solutions for each compound (HGR4113 and HGR4113-d7) and the IS (HSG4112) were formulated in ACN at a concentration of 1 mg/mL. Then, working solutions with a range of 0.2–160 µg/mL for HGR4113 and 0.1–20 µg/mL for HGR4113-d7 were prepared after serial dilutions. The calibration and QC standards were constructed on working solutions (5 µL) by spiking the analyte into blank rat plasma (95 µL). Calibration standards were formulated with concentrations ranging from 10 to 8000 ng/mL (10, 20, 50, 100, 250, 500, 1000, 4000, and 8000 ng/mL) for HGR4113 and from 5 to 1000 ng/mL (5, 10, 25, 50, 100, 250, 500, and 1000 ng/mL) for HGR4113-d7. Different concentrations of QC samples at the lower limit of quantification (LLOQ) and at low, middle, and high concentrations of 10, 30, 800, and 6000 ng/mL for HGR4113 and 5, 15, 200, and 800 ng/mL for HGR4113-d7, respectively, were prepared. The IS stock solution was adjusted with ACN to prepare a concentration of 600 ng/mL.

### 2.6. Plasma Sample Preparation

Plasma (20 µL) was collected in Eppendorf tubes (1.5 mL), and then 80 μL of HSG4112 (IS, 600 ng/mL) was applied. The tube was centrifuged for 5 min at 13,000× *g* at 4 °C after being vortexed for one minute. Subsequently, the supernatant was collected in an LC vial to be analyzed via LC-MS/MS.

### 2.7. In Vitro Microsomal Metabolic Stability

HGR4113 and HGR4113-d7 (final concentration of 5 µM) were pre-incubated with 1 mg/mL of rat liver microsomes (RLM) in 0.1 M potassium phosphate buffer (pH 7.4) at a temperature of 37 °C for 5 min. To initiate the assay, an NADPH-generating system solution comprising 10 mg/mL β-NADP+, 1 unit/mL of glucose-6-phosphate dehydrogenase, and 0.1 M glucose-6-phosphate was mixed to make the final volume of 200 μL (n = 3). Each reaction was terminated at 0, 5, 15, 30, 60, and 90 min by adding 400 μL of ACN. Then, the samples were centrifuged for 5 min at 13,000× *g*, and the supernatant was analyzed using LC-MS/MS.

### 2.8. LC-MS/MS

An Acquity UPLC with tandem mass spectrometry (Waters, Milford, MA, USA) and its electrospray ionization mode was utilized. Spectrometric detection was carried out in negative ion mode. For separation, the Halo^®^ C18 column (2.1 × 100 mm, 2.7 µm) was utilized. The mobile phase (solvent A) comprised 0.1% acetic acid in DW and 0.1% acetic acid in ACN: MeOH = 3:1 (*v*/*v*) (solvent B). To quantify the analytes, gradient elution was carried out at a speed of 0.2 mL/min (0.0–0.8 min, 10% B; 0.9–4.1 min, 90% B; 4.2–7.0 min, 10% B). The target ions used were *m*/*z* 367.2→177.1 for HGR4113, *m*/*z* 374.2→177.1 for HGR4113-d7, and *m*/*z* 353.3→137.0 for HSG4112 (IS), using multiple reaction monitoring (MRM) analyses. The cone voltage was set to 55 V, and the capillary voltage was set to 3 kV, while the collision energy (CE) was 27 V. Nitrogen (desolvation gas) was used at a speed of 600 L/h and at a fixed temperature of 450 °C.

### 2.9. Method Validation

Method selectivity was measured by comparing blank rat plasma with six individual sources and LLOQ standard-spiked plasma samples for endogenous interference.

Linearity was evaluated by the coefficient of determination (R^2^) of the calibration curves with concentrations ranging from 10 to 8000 ng/mL for HGR4113 and from 5 to 1000 ng/mL for HGR4113-d7. The calibration range was designated by the lowest calibration standard (i.e., LLOQ) and the upper limit of quantification (the highest calibration standard). Using this calibration curve, the calibration standard’s back-calculated concentration was calculated from triplicate analyses.

Accuracy and precision were investigated by measuring four concentrations of QC samples—LLOQ and low, middle, and high—corresponding to 10, 30, 800, and 6000 ng/mL for HGR4113 and 5, 15, 200, and 800 ng/mL for HGR4113-d7, respectively. In both intra- and inter-day batches, five samples per concentration of each QC sample were analyzed for 3 days. Intra-day precision calculation was indicated as the coefficient of variation (CV) for the concentration observed in the sample analyzed on the same day. The intra-day accuracy is calculated by the comparison of samples’ average experimented concentration and their theoretical values. The intra-day accuracy and precision were calculated using the same methodology.

The process efficiency, extraction recovery, and the matrix effect were evaluated at three QC concentrations: low, medium, and high. The peak area of the pre-spiked extracted plasma sample was compared to the post-spiked extracted sample in order to assess extraction recovery. Associating the peak area of tidy solution with the pre-spiked retrieved sample allowed us to assess the process efficiency. Matrix effects were examined by measuring the peak area in the post-spiked extracted sample compared to a clean solution.

Stability studies at low- and high-QC concentrations were investigated for freeze–thaw, short-term, long-term, and processed plasma sample stability. For QC samples, after 12 h of freezing at a temperature of −20 °C and thawing at 20–25 °C for the next 12 h in cycles repeated three times, they were tested for freeze–thaw stability. The QC samples were retained at 20–25 °C for 8 h for the short-term stability tests. QC samples were stored at −20 °C for a month as part of the long-term stability test, and they were placed in an auto-sampler for 24 h at 4 °C to test the stability of the processed samples.

### 2.10. Data Analysis

Pheonix WinNonlin (Ver. 6.2, Certara, Princeton, NJ, USA) was employed to calculate pharmacokinetic parameters based on non-compartmental model. The oral bioavailability (F) of HGR4113 was calculated as follows [23]:

F = [(AUC_oral_ × Dose_intravenous_)/(AUC_intravenous_ × Dose_oral_)] × 100 (1)

The significance of pharmacokinetic values was measured by paired Student’s *t*-test. A probability value (*p*-value) of <0.05 was assumed statistically significant. For variance, one-way ANOVA was applied to compare the bioavailability of individual rats in whole groups.

## 3. Results

### 3.1. Bioanalytical Method Validation

**Figure 2:**
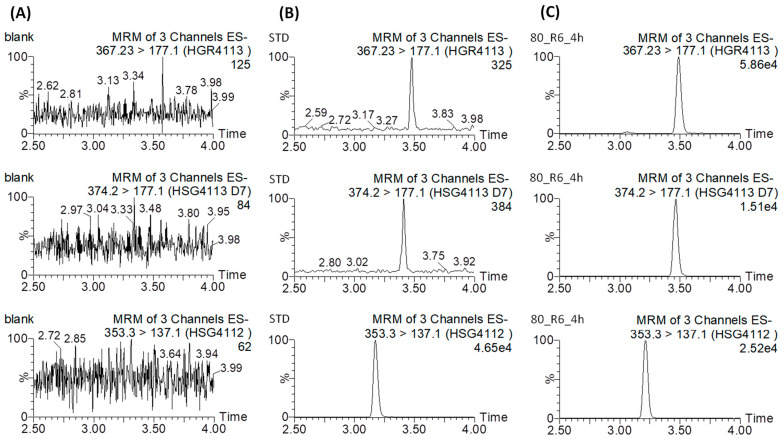
Representative extracted ion chromatograms of HGR4113 (**upper**), HGR4113-d7 (**middle**), and HSG4112 (internal standard) (**bottom**) in (**A**) blank rat plasma, (**B**) blank rat plasma spiked with HGR4113 and HGR4113-d7 standards at LLOQ, and (**C**) the plasma sample collected at 6 h after oral administration.

Selectivity was assessed by way of comparing the rat blank plasma sample and the rat blank plasma sample spiked with the QC sample at the LLOQ level. The representative MRM chromatograms are shown in Figure 2. No endogenous interference was noted as peak areas were >20% of the LLOQ at the analyte or IS retention time. Over the entire calibration range, the calibration curves have acceptable linearity with respect to the standard concentrations, and the regression coefficients (R^2^) were ≥0.99 (Table S1). The intra- and inter-day precisions and accuracies are presented in Table 1. The intra- and inter-day accuracies were within ±15%, while their precision was <15% CV. The matrix effect, extraction recovery, and process efficiency results are listed in Table 2. The mean matrix effect ranged from 76.3 to 95.0 for HGR4113 and from 109.0 to 114.3 for HGR4113-d7. The average recovery ranged from 70.2 to 97.1 for HGR4113 and from 103.2 to 117.9 for HGR4113-d7, and the process efficiency ranged from 87.5 to 97.5 for HGR4113 and from 94.0 to 108.3 for HGR4113-d7. The freeze–thaw stability, short-term and long-term stability, and processed sample stability were tested. All four stability test results satisfied the required criteria (accuracy < ±15%; precision < ±15% CV; Table S2).

### 3.2. Pharmacokinetic Equivalence of HGR4113 and HGR4113-d7

**Figure 3:**
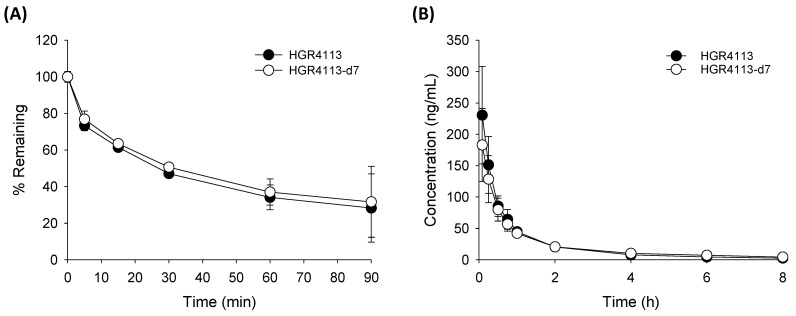
Pharmacokinetic equivalency of HGR4113 and HGR4113-d7. (**A**) Liver microsomal metabolic stability. HGR4113 and HGR4113-d7 were incubated with rat liver microsomes at 5 μM, and the remaining amounts of HGR4113 and HGR4113-d7 at each time point were determined (n = 3). (**B**) Plasma concentration–time curves of HGR4113 and HGR4113-d7 after IV administration. Rats were intravenously administered a 1 mg/kg mixture of HGR4113:HGR4113-d7 (1:1), and the plasma concentrations of HGR4113 and HGR4113-d7 were determined (n = 4).

The metabolic stability of HGR4113 and HGR4113-d7 in RLM is shown in Figure 3A. The remaining amounts of HGR4113 and HGR4113-d7 at 90 min were 28.3% ± 18.6% and 31.7% ± 19.4%, respectively, indicating a non-significant statistical difference between the two groups. Plasma concentration–time plots of HGR4113 and HGR4113-d7 after the IV injection of a 1:1 mixture of HGR4113 and HGR4113-d7 are reflected in Figure 3B. Pharmacokinetic parameters of HGR4113 and HGR4113-d7 are listed in Table 3. As a result of the paired Student’s *t*-test on the pharmacokinetic parameters between the two groups, there was non-significant variation in the AUC and other pharmacokinetic values except for MRT.

### 3.3. Bioavailability of HGR4113

**Figure 4:**
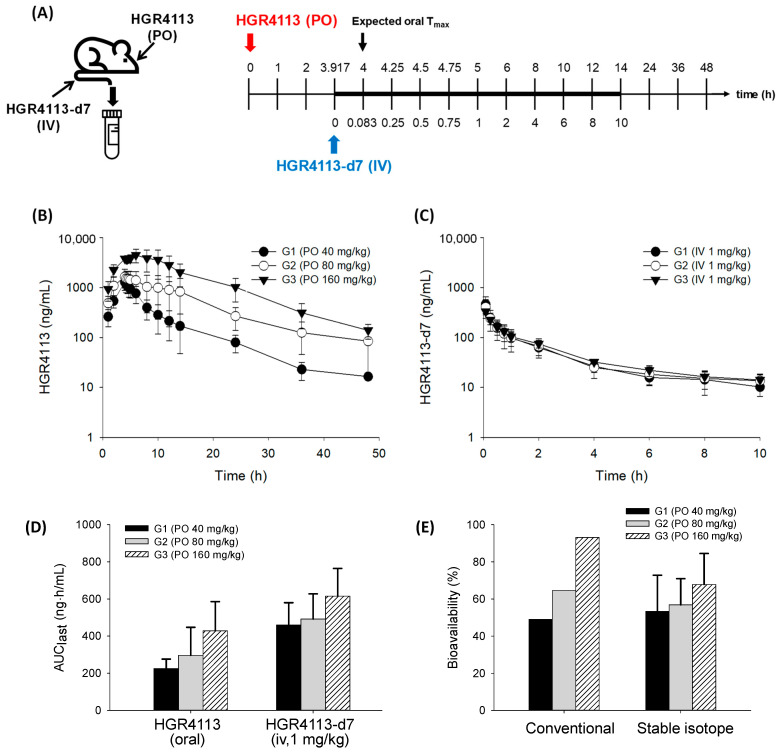
Evaluation of bioavailability of HGR4113. (**A**) Experimental schedule for drug administration and blood sample collection. (**B**) Plasma concentration–time curves of HGR4113 after oral administration in G1 (40 mg/kg, n = 5), G2 (80 mg/kg, n = 6), and G3 (160 mg/kg, n = 6). (**C**) Plasma concentration–time curves of HGR4113-d7 after iv administration at a dose of 1 mg/kg to G1, G2, and G3 rats. (**D**) Area under curve (AUC) values of HGR4113 and HGR4113-d7. (**E**) Oral bioavailability of HGR4113 in each dose group. For the conventional method, the mean AUC value of HGR4113-d7 taken from G1 was commonly applied to the mean AUC values of three oral doses, and, for the stable isotope method, the bioavailability was calculated by the comparison of the AUC of HGR4113 (oral) with HGR4113-d7 (intravenous) for each individual rat.

The profiles of plasma concentrations versus time of HGR4113 and HGR4113-d7, administering an oral dose of HGR4113 at 40 (G1), 80 (G2), and 160 (G3) mg/kg and with an IV dose of HGR4113-d7 (1 mg/kg), are displayed in Figure 4B,C. Based on their levels in plasma, we parameterized the pharmacokinetic characteristics of each compound (Table 4). The oral bioavailability of HGR4113 was calculated in two ways: (1) the conventional way in which it is assumed that the mean AUC of iv at one fixed dose (the AUC data of HGR4113-d7 was taken from the G1 group) was commonly applied to the three oral doses and (2) our proposed method (the stable isotope method) in which the bioavailability was calculated by the comparison of the AUC of HGR4113 (oral) with HGR4113-d7 (intravenous) for each individual rat. The oral bioavailability of HGR4113 was calculated to be 49.1%, 64.5%, and 93.1% by the conventional method and 53.3%, 56.9%, and 67.8% by the stable isotope method at 40, 80, and 160 mg/kg doses, respectively (Figure 4E).

## 4. Discussion

In this study, the bioavailability of HGR4113 in rats was evaluated with a stable isotope tracer (HGR4113-d7) and LC-MS/MS. HGR4113 shows poor water solubility and is a good model for stable isotope tracer testing. The HGR4113-d7 was intravenously administered to rats that were orally pre-administered HGR4113 at different doses. Subsequently, HGR4113 and HGR4113-d7 were simultaneously determined in the plasma samples, and bioavailability was calculated. The bioavailability of HGR4113 was 53.3%~67.8% after oral dosages of 40, 80, and 160 mg/kg, respectively. The current method reduced measurement errors in bioavailability by eliminating the differences in clearance between intravenous and oral dosages compared to the conventional approach.

Stable isotopes such as ^13^C, ^2^H, ^15^N, and ^18^O have been used to evaluate absolute bioavailability in clinical pharmacokinetics [24,25,26,27,28,29,30,31,32,33,34,35]. Among these, ^2^H (deuterium) has been widely used owing to its ease of synthesis [16]. Deuterium-labeled drugs, however, may exhibit kinetic isotope effects [36,37,38]. Accordingly, prior to the evaluation, the pharmacokinetic equivalence of HGR4113 and HGR4113-d7 was confirmed by comparing their metabolic stability and in vivo pharmacokinetic profiles; the results indicated that the kinetic isotope effect of HGR4113-d7 was negligible (Figure 3).

In the stable isotope tracer method, the resemblance in the shape of the plasma concentration–time profile between orally and intravenously dosed drugs during their absorption phase is likely to indicate the similarity of their clearance. To this end, Rubin et al. suggested that the intravenous stable isotope tracer dose should be administered either at T_max_ or at the time delayed by half of T_max_ [16,39] of the oral dose rather than administered simultaneously. In our previous study, orally administered HGR4113 reached the maximum plasma concentration at 2.3–4.4 h with 10–100 mg/kg doses (Table S3). Based on this, HGR4113-d7 was administered intravenously 3 h and 55 min after oral HGR4113 administration.

The oral bioavailability of HGR4113 was estimated to be 53.3% ± 19.5%, 56.9% ± 14.0%, and 67.8% ± 16.7% after the administration of 40, 80, and 160 mg/kg doses, respectively. The statistical analysis showed no significant differences between the three groups. Nevertheless, a gradual increase in bioavailability with increasing doses implied there might be saturation in the clearance pathways of HGR4113 at higher doses. It was notable that the AUC values of intravenous HGR4113-d7, even at the same dose (1 mg/kg), differed according to the oral dose of the corresponding groups. The AUC values of HGR4113-d7 were shown to be 0.46, 0.49, and 0.62 μg·h/mL in G1 (40 mg/kg), G2 (80 mg/kg), and G3 (160 mg/kg), respectively. This indicates that if oral bioavailability is calculated by applying identical intravenous AUC values irrespective of oral doses, as measured in the conventional preclinical study, the oral bioavailability differences among the three groups would significantly widen (Figure 4E). Such differences in oral bioavailability in different doses were observed in data obtained using the conventional method (i.e., intravenous and oral administrations of HGR4113 in separated groups). The oral bioavailability of HGR4113 was calculated to be 13.6%, 38.0%, and 85.0% at the administration of 10, 30, and 100 mg/kg doses, respectively (Table S3). A considerable variance (13%~85%) in the oral bioavailability of each dose was observed. Thus, oral bioavailability could be different depending on the dose at which the bioavailability is calculated. Such variance could misleadingly offer the wrong estimation of bioavailability in humans. Even considering the difference in the dose ranges investigated between the previous and current studies, it is strongly suggested that the previous data could be distorted. The present method may correct such bias, which is possibly caused by different clearance profiles between intravenous and oral doses.

Up to now, methods using radiolabeled drugs with accelerator mass spectrometry have been widely adopted to evaluate human oral bioavailability studies instead of using stable isotopes in combination with LC-MS/MS due to sensitivity issues. However, recent technical progress in mass spectrometry has enabled the detection of low-dose intravenous drugs and, thus, may revive the application of stable isotopes to evaluate the preclinical bioavailability. The advantages of stable isotopes (e.g., low cost, synthesis ease, handling convenience, detection methods, and safety) could minimize the time and effort needed for drawing the bioavailability of poorly water-soluble drugs.

## 5. Conclusions

This study demonstrates the potential of stable isotopes for the reliable assessment of the bioavailability of poorly water-soluble drugs, using HGR4113 as a model drug. Compared to traditional methods, the proposed strategy significantly reduces the discrepancy in bioavailability by rectifying the variance from the different clearance profiles between intravenous and oral doses at different levels. This method could be widely applied to assess the bioavailability of new drug candidates in the preclinical stage, thereby increasing their chances of successful clinical stage entry.

## 6. Recommendations

The escalating number of newly developed drug candidates possessing low aqueous solubility has posed a challenge in accurately assessing their bioavailability by conventional methods. Furthermore, our research revealed substantial variation in the bioavailability of HGR4113 from its actual value when conventional methods were utilized. Therefore, we strongly recommend adapting our proposed method for assessing the bioavailability of poorly water-soluble drugs, which can avert any misinterpretations that could adversely impact their success in clinical application.

## Figures and Tables

**Table 1:** Intra-day and inter-day accuracy and coefficient of variation for determination of HGR4113 and HGR4113-d7 in rat plasma.

Compound	Nominal Concentration (ng/mL)	Intra-Run (n = 5)		Inter-Run (n = 5)	
		Accuracy (%)	CV (%)	Accuracy (%)	CV (%)
HGR4113	10	100.6 ± 9.0	8.9	93.8 ± 11.4	12.1
	30	93.3 ± 9.2	9.8	95.6 ± 9.1	9.5
	800	107.9 ± 2.9	2.7	110.3 ± 3.4	3.1
	6000	91.6 ± 2.8	3.1	90.3 ± 2.7	3.0
HGR4113-d7	5	108.0 ± 6.6	6.1	101.9 ± 11.2	11.0
	15	100.5 ± 4.2	4.2	98.0 ± 6.2	6.3
	200	107.7 ± 2.6	2.4	100.6 ± 6.2	6.1
	800	107.1 ± 3.4	3.2	104.6 ± 3.6	3.4

**Table 2:** Matrix effect, recovery, and process efficiency data for HGR4113 in rat plasma (n = 3).

Compound	Nominal Concentration (ng/mL)	Matrix Effect (%)	Recovery (%)	Process Efficiency (%)
HGR4113	30	76.3 ± 3.2	70.2 ± 7.2	87.5 ± 13.9
	800	95.0 ± 3.3	97.1 ± 5.9	97.5 ± 2.9
	6000	90.1 ± 2.3	82.0 ± 2.3	91.7 ± 1.0
HGR4113-d7	15	109.0 ± 12.1	117.9 ± 13.3	108.3 ± 7.0
	200	109.9 ± 7.0	103.2 ± 8.1	94.0 ± 6.3
	800	114.3 ± 5.4	109.2 ± 5.2	95.6 ± 2.7

**Table 3:** Pharmacokinetic parameters of HGR4113 and HGR4113-d7 after IV administration of mixture 1 mg/kg (n = 4).

PK Parameter	HGR4113	HGR4113-d7
AUC_0-t_ (μg·h/mL)	0.18 ± 0.02	0.18 ± 0.02
T_1/2_ (h)	2.39 ± 1.04	3.60 ± 1.63
AUC_0-∞_ (μg·h/mL)	0.19 ± 0.03	0.20 ± 0.03
MRT_last_ (h)	1.41 ± 0.43	1.79 ± 0.29 *
CL (L/h/kg)	2.64 ± 0.35	2.51 ± 0.37
Vz (L/kg)	8.82 ± 3.32	12.44 ± 4.05

The data are presented as the mean with standard deviation. Vz, Volume of distribution; AUC, area under the plasma concentration–time-curve; T_1/2_, terminal half-life; CL, total body clearance; MRT, mean residence time. *: *p* < 0.05 versus HGR4113.

**Table 4:** Pharmacokinetic parameters of HGR4113 and HGR4113-d7 in rats.

PK Parameter	G1 (n = 5)		G2 (n = 6)		G3 (n = 6)	
	IV 1 mg/kg	PO 40 mg/kg	IV 1 mg/kg	PO 80 mg/kg	IV 1 mg/kg	PO 160 mg/kg
T_max_ (h)	-	4.00 ^b^	-	5.00 ^b^	-	6.00 ^b^
C_max_ (μg/mL)	-	1.29 ± 0.40	-	1.79 ± 0.73	-	4.90 ± 1.37
AUC_0−t_ (μg·h/mL)	0.46 ± 0.12	9.02 ± 2.02	0.49 ± 0.14	23.73 ± 12.03	0.62 ± 0.15	68.49 ± 25.25
T_1/2_ (h)	3.23 ± 0.92	9.28 ± 1.06	5.40 ± 4.04	13.97 ± 8.64	7.91 ± 3.38	7.75 ± 1.03
AUC_0-∞_ (μg·h/mL)	0.51 ± 0.13	9.23 ± 2.03	0.57 ± 0.18	26.07 ± 12.39	0.69 ± 0.16	70.09 ± 25.33
MRT_last_ (h)	2.32 ± 0.43	9.18 ± 2.10	3.51 ± 3.25	13.11 ± 4.03	4.76 ± 1.55	12.66 ± 2.02
Cl (L/hr/kg)	2.15 ± 0.71	-	1.93 ± 0.62	-	1.52 ± 0.37	-
Vz (L/kg)	10.38 ± 5.61	-	13.12 ± 9.13	-	15.95 ± 6.02	-
F (%) ^a^	53.34 ± 19.46		56.88 ± 14.03		67.80 ± 16.70	
*p*-value ^c^	0.411 (>0.05)					

The data are presented as the mean with standard deviation. C_max_, Maximum experimented plasma concentration; T_max_, time for C_max_ occurrence; Vz, volume of distribution; AUC, area under the plasma concentration–time curve; T_1/2_, terminal half-life; CL, total body clearance; MRT, mean residence time. ^a^ Bioavailability was calculated for each individual rat. ^b^ The data are presented as the median. ^c^ *p*-value was calculated by comparing the bioavailability (F (%)) of G1 to G3 using one-way ANOVA.

## Data Availability

Not applicable.

## Supplementary Materials

The following supporting information can be downloaded at https://www.mdpi.com/article/10.3390/pharmaceutics15061684/s1. Figure S1: Study flow chart; Figure S2: Chemical structures of (A) HGR4113, (B) HGR4113-d7, and (C) HSG4112 (IS); Table S1: Results of HGR4113 and HGR4113-d7 linearity in rat plasma (n = 3); Table S2: Stability of HGR4113 and HGR4113-d7 in rat plasma (n = 3); Table S3: Pharmacokinetic parameters of HGR4113 after IV administration of 1 mg/kg (n = 5) and PO administration of 10, 30, and 100 mg/kg (n = 5).
