# Supplementary material for: Preclinical Bioavailability Assessment of a Poorly Water-Soluble Drug, HGR4113, Using a Stable Isotope Tracer
Source: Pharmaceutics. 2023 Jun 8;15(6):1684. doi: 10.3390/pharmaceutics15061684 (PMC10305361; doi:10.3390/pharmaceutics15061684)
Supplement: Supplementary file 1 [file pharmaceutics-15-01684-s001.zip › pharmaceutics-2402646-supplementary.pdf]

Assessment of the bioavailability of a poorly water-soluble drug, HGR4113 using a stable isotope tracer for preclinical drug development

Eun Ji Ha[1, †], Jeong In Seo[1, †], Shaheed Ur Rehman[2], Hyung Soon Park[3], Sang-Ku Yoo[3], Hye Hyun Yoo[1,*]

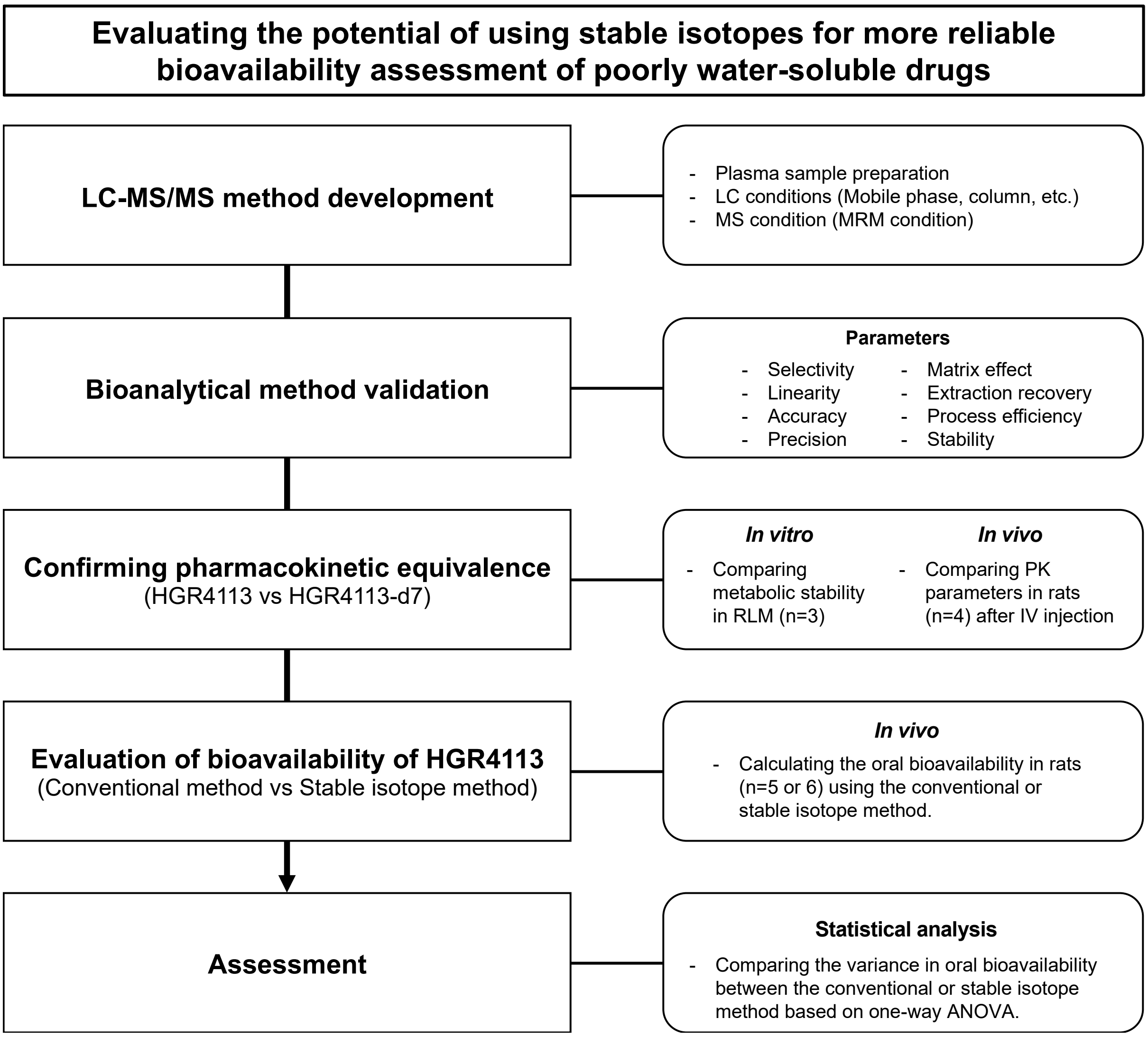

Figure S1. Study flow chart

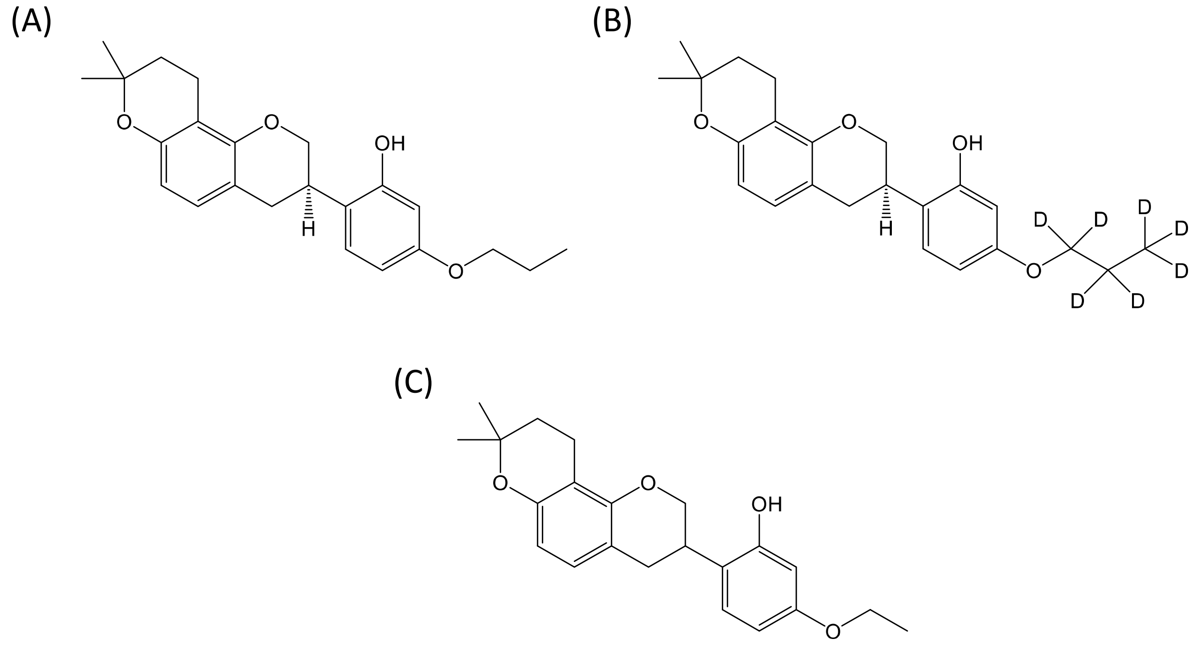

Figure S2. Chemical structures of (A) HGR4113, (B) HGR4113-d7 and (C) HSG4112 (IS)

Table S1. Results of HGR4113 and HGR4113-d7 linearity in rat plasma (n=3)

Compound	Nominal concentration (ng/mL)	Measured concentration (ng/mL)	Regression equation	Accuracy (%)	Precision (%)
HGR4113	10	10.3±0.2	m*=0.0014 ±0.0000	102.7	1.7
	20	19.3±1.1		96.3	4.8
	50	46.5±5.9		93.1	10.3
	100	102.6±3.2	b**=-0.0024 ±0.0023	102.6	2.5
	250	253.7±11.3		101.5	3.7
	500	548.6±12.0		109.7	1.8
	1000	1082.9±36.6	R^2***=0.9917 ±0.0001	108.3	2.8
	4000	3709.8±109.1		92.7	2.5
	8000	7452.7±562.6		93.2	6.2
HGR4113-d7	5	5.1±0.2	m*=0.0015 ±0.0001	101.3	4.1
	10	10.1±0.8		100.7	7.8
	25	23.5±0.6		93.9	2.5
	50	48.8±1.6	b**=-0.0026 ±0.0011	97.6	3.3
	100	101.9±4.8		101.9	4.7
	250	267.4±7.9		107.0	3.0
	500	534.1±5.9	R^2***=0.9934 ±0.0012	106.8	1.1
	1000	914.4±20.3		91.4	2.2

*: m=slope, **: b=y-intercept, ***: R^2=linear regression coefficient

Table S2. Stability for HGR4113 and HGR4113-d7 in rat plasma (n=3)

**Compound**	**Storage condition**	**Nominal concentration (ng/mL)**	**Measured concentration (ng/mL)**	**Accuracy (%)**	**CV (%)**
HGR4113	Freeze and thaw	30	29.5±1.7	98.4	5.9
		6000	5920.9±276.7	98.7	4.7
	Short term	30	30.8±3.3	102.8	10.8
		6000	5444.3±140.2	90.7	2.6
	Long term	30	30.4±0.8	101.3	2.6
		6000	6122.5±160.1	102.0	2.6
	Processed sample	30	27.5±0.9	91.7	3.3
		6000	6102.2±171.7	101.7	2.8
HGR4113-d7	Freeze and thaw	15	14.7±1.0	97.8	6.6
		800	901.3±8.3	112.7	0.9
	Short term	15	13.5±0.3	90.2	2.4
		800	886.4±3.4	110.8	0.4
	Long term	15	14.6±1.1	97.1	7.8
		800	845.5±24.6	105.7	2.9
	Processed sample	15	14.8±0.7	98.7	4.8
		800	896.8±22.3	112.1	2.5

Table S3. Pharmacokinetic parameters of HGR4113 after IV administration of 1 mg/kg (n=5) and PO administration of 10, 30 and 100 mg/kg (n=5)

PK Parameter	IV 1 mg/kg	PO 10 mg/kg	PO 30 mg/kg	PO 100 mg/kg
**T_{max} (h)**	0.12±0.07	2.21±1.59	3.60±1.50	4.40±0.80
**C_{max} (μg/mL)**	0.13±0.02	0.08±0.04	0.56±0.27	2.27±0.55
**AUC_{0-t} (μg·h/mL)**	0.26±0.04	0.36±0.16	3.00±1.70	22.38±6.36
**$T_{1/2}$ (h)**	2.08±0.88	4.37±1.55	2.63±1.14	6.04±1.33
**$AUC_{0-\infty}$ (μg·h/mL)**	0.28±0.03	0.42±0.16	3.03±1.70	24.67±7.21
**MRT_{last} (h)**	2.08±0.24	3.99±0.65	4.74±1.03	7.96±0.97
**Cl (L/hr/kg)**	3.63±0.42			
**Vz (L/kg)**	11.9±5.74			
**F (%)**		13.64	37.96	84.99
